# Sex differences in mood disorders: perspectives from humans and rodent models

**DOI:** 10.1186/s13293-014-0017-3

**Published:** 2014-12-07

**Authors:** Marianne L Seney, Etienne Sibille

**Affiliations:** Department of Psychiatry, Translational Neuroscience Program, University of Pittsburgh, Pittsburgh, PA 15213 USA; Center for Neuroscience, University of Pittsburgh, Pittsburgh, PA 15213 USA; Departments of Psychiatry, Campbell Family Mental Health Research Institute, Centre for Addiction and Mental Health, Pharmacology and Toxicology, University of Toronto, Toronto, ON M5T 1R8 Canada

**Keywords:** Major depressive disorder, Sex difference, Corticolimbic, Somatostatin, Four core genotypes (FCG), Gamma-aminobutyric acid (GABA)

## Abstract

Mood disorders are devastating, often chronic illnesses characterized by low mood, poor affect, and anhedonia. Notably, mood disorders are approximately twice as prevalent in women compared to men. If sex differences in mood are due to underlying biological sex differences, a better understanding of the biology is warranted to develop better treatment or even prevention of these debilitating disorders. In this review, our goals are to: 1) summarize the literature related to mood disorders with respect to sex differences in prevalence, 2) introduce the corticolimbic brain network of mood regulation, 3) discuss strategies and challenges of modeling mood disorders in mice, 4) discuss mechanisms underlying sex differences and how these can be tested in mice, and 5) discuss how our group and others have used a translational approach to investigate mechanisms underlying sex differences in mood disorders in humans and mice.

## Review

### Sex differenced in major depression

Major depressive disorder (MDD) is a severe mental illness and the leading cause of disability and of years of productivity lost worldwide [1]. In addition to the psychological stress on patients and families, MDD contributes to the development and progression of systemic and organ diseases [2-5]. For instance, MDD increases the risk for coronary heart disease incidence by approximately 1.7 times compared to non-depressed subjects [6], and MDD patients have a 37% increased risk for developing type 2 diabetes [7]. Moreover, patients with mood disorders (MDD or bipolar disorder) make up approximately 60% of completed suicides [8]. MDD is defined as a syndrome that includes prominent emotion dysregulation, low mood, poor affect, and/or anhedonia; these core MDD symptoms are accompanied by cognitive symptoms (attention, concentration), physiological symptoms (weight, locomotor, and sleep pattern changes) [9], and frequent co-morbid high anxiety symptoms [9,10].

Notably, women are twice as likely to be diagnosed with MDD compared to men [11,12]. When men and women that have been diagnosed with MDD are compared, women tend to have more symptoms and higher symptom severity, and women report more subjective distress [13-15]. Additionally, anxiety symptoms are almost always co-morbid with MDD in women, making the two difficult to separate. In fact, women are more likely than men to have a co-morbid anxiety disorder with MDD (e.g., [16]), and men more likely to have a co-morbid substance abuse disorder (reviewed in [17]), possibly suggesting different coping strategies in males and females. A frequent and important question is whether the sex difference in MDD incidence is an artifact of women being more likely to seek treatment. However, this sex difference in MDD incidence is consistently found across cultures and in community-based epidemiological studies, in which the factor of seeking treatment is removed (e.g., [13,18]), suggesting that there are biological differences that place women at increased risk for MDD. Also arguing against the potential artifact of women being more likely to seek treatment, Bogner and Gallo found no sex difference in self-report of depressive symptoms in a community-based epidemiological study [19].

Some studies suggest that women respond differently than men to antidepressant treatment. For instance, a study by Kornstein et al. [11] found that women responded more favorably (i.e., reduced symptoms, fewer adverse effects) to selective serotonin reuptake inhibitors (SSRIs) than men; conversely, men responded more favorably than women to tricyclic antidepressants [11,20]. However, other studies have reported no sex differences in response to SSRIs or tricyclics (e.g., [21]), but a statistically superior response to monoamine oxidase inhibitors (MAOIs) in women compared to men [21]. Entsuah et al. [22] found no sex difference in response to SSRIs or to venlafaxine, a serotonin norepinephrine reuptake inhibitor. Taken together, this suggests variable or no sex differences in antidepressant response, specifically compared to the robust and replicated findings of sex differences in symptom dimensions.

It has been proposed that the increased prevalence of MDD in women may be due to how women perceive stress [23]. In other words, women may have the “trait” of having more subjective distress in stressful situations compared to men. Indeed, even when considering men and women without an MDD diagnosis, there are sex differences in response to stressful situations. For instance, even when men and women have equivalent physiological responses to the same stressful situation (no differences in heart rate or plasma cortisol), women self-report higher irritability and fear as well as decreased happiness compared to men [23].

### Depression-related sex differences in a corticolimbic network of mood regulation

Even though the neurobiological mechanism(s) underlying MDD remain poorly characterized, evidence from both neuroimaging and postmortem neuroanatomical and molecular studies suggest a dysfunction in the emotion regulation centers of the brain underlying low affect, a symptom dimension common to both MDD and anxiety disorders [24-27]. This corticolimbic network includes the prefrontal and anterior cingulate cortices, the hippocampus, the anterior thalamic nuclei, and the amygdala [24,28]. The subgenual anterior cingulate cortex (sgACC) consistently shows elevated metabolic activity with the induction of depressive states [29-31], which returns to normal following antidepressant treatment [30] or deep brain stimulation [32]. Interestingly, neuroimaging studies show that features of sgACC dysfunction in MDD are sexually dimorphic, with women exhibiting higher levels of reactivity compared to males [33-35]. The amygdala processes emotionally salient stimuli and, in concert with cortical and subcortical interconnections, initiates a behavioral response [27]. Neuroimaging studies show that MDD patients exhibit abnormal processing of emotional stimuli, with sustained amygdala reactivity [36,37] (although [38,39]). Similarly, amygdala hyperactivity is reported in patients with various anxiety disorders, including post-traumatic stress, generalized anxiety, and social anxiety disorders [40].

### How do we model mood disorders in mice?

#### Validity of animal models

When assessing any animal model of a psychiatric disorder, several criteria need to be considered. The animal model should have construct validity, that is, it should follow a similar etiology as the human disorder. The model should have face validity, with anatomical, behavioral, or molecular features of the disorder being replicated. Predictive validity should also be considered, as pharmacological treatment in the animal model should recapitulate the effects of treatment in humans. Importantly, both the effect and time-course of efficacious treatments in humans need to be taken into account when assessing predictive validity of an animal model.

#### Trait versus state

When researchers investigate anxiety-/depressive-like behavior in mice, they often do so under baseline (i.e., “trait”) conditions. These traits represent properties of the biological and behavioral system that may play a role in susceptibility to develop a psychiatric disorder. On the other hand, MDD represents a temporary mood “state”; in other words, MDD can be considered a transient pathological state that is brought on by certain factors, i.e., depressive episodes. Studies under baseline conditions are relevant, as they can provide insight into potential predisposition for developing an MDD state. We argue, however, that many studies only examine trait conditions. We feel that this is especially true in mouse studies investigating the origin of sex differences in anxiety-/depressive-like behaviors. There are, however, models that researchers can use to examine these anxiety-/depressive-like behaviors under pathological state conditions that are homologous to depressive episodes.

One model that is used to study mice in an elevated mood-related state is unpredictable chronic mild stress (UCMS). UCMS was originally developed in rats, and our lab and others have recently used UCMS in mice to model human MDD episodes. UCMS replicates the role of stress in eliciting MDD, with rodents developing a depressive-like syndrome after several weeks of random exposure to mild social and environmental stressors. Specifically, these mice have heightened fearfulness/anxiety-like behavior [41], anhedonia-like behavior, as assessed by decreased consumption of palatable food and drink [41,42] and decreased sensitivity to rewards [43], and physiological symptoms (decreased weight gain and grooming behavior (e.g., [44]). Additionally, there is dysregulation of the hypothalamic pituitary adrenal (HPA) axis and elevated basal plasma corticosterone [45], as reported in some MDD patients (e.g., [46-49]). The UCMS syndrome respects the time frame of onset and efficacy of antidepressant treatment [42,50,51]. Interestingly, not all mice exhibit a depressive-like syndrome following UCMS exposure, making it more realistic, as differences in response to stress exposure are also observed in humans (e.g., [52]), and making it a potential model to study both vulnerability and resiliency to develop a depressive-like episode. One significant drawback of UCMS is that it is not a simple procedure to perform: it is labor intensive and lasts for 4–9 weeks. Additionally, UCMS is not as highly reproducible as some other mouse models of MDD (e.g., genetic models), and this may be due to a number of factors, including among others varying stress procedures, duration of UCMS, strain of mice used, and normal heterogeneity in stress response.

Another mouse model that elicits an elevated mood state is chronic social defeat stress. With this paradigm, male rodents are subjected to repeated bouts of social subordination [53]. There are several benefits of this paradigm: 1) it has construct validity (chronic stress elicits the behavioral deficits), 2) it has predictive validity (chronic antidepressant treatment reverses behavioral deficits), 3) it affects multiple systems as MDD does (e.g., dopaminergic reward circuits and hippocampal neurotrophin), and 4) it is useful to study mechanisms underlying resilience (e.g., [54]). However, there is a challenge with the chronic social defeat paradigm when one is interested in sex differences in MDD, since this paradigm seems to only be effective in male, but not female C57BL/6 mice. Researchers have got around this limitation by using different species of mice or by using rats (e.g., [55-57]). The social defeat paradigm has been used successfully in the monogamous California mouse (*Peromyscus californicus*), in which both males and females aggressively defend territories [58]. Interestingly, Trainor and colleagues [59] reported no effect of adult hormone manipulation in the paradigm, but an effect of corncob bedding (which has estrogenic properties) during development, together suggesting developmental hormonal programming.

Learned helplessness is another model used in the rodent literature to induce a depressive-like state [60,61]. In this model, the rodent is exposed to a noxious stimulus (often a shock) that it either can or cannot escape. When later tested under conditions in which escape is possible, the rodent that previously was exposed to the inescapable shock often does not learn to escape. Importantly, the learned helplessness model has: 1) construct validity (uncontrollable stressful events precipitate the deficit), 2) predictive validity (improved response after antidepressant treatment), and 3) face validity (equated with the helplessness experienced by humans with MDD). A very interesting aspect of the learned helplessness model is that it seems to be ineffective in eliciting a depressive-like state in female rats [62] and in female C57BL/6 mice [63]. This sex difference may be strain/species specific, as both male and female 129SvEv mice develop learned helplessness [63]. Notably, the sex difference in learned helplessness in rats was not reversed after removal of adult hormones by gonadectomy [62], suggesting either developmental hormonal or sex chromosome complement effects.

### Modeling sex differences in mice

When a sex difference is observed, there are several steps that can be taken to determine the cause(s) of the sex difference. There are several comprehensive reviews on this topic (e.g., [64-66]); we summarize the general strategy here. The first and easiest step is to test whether the sex difference disappears after normalizing, or “clamping”, circulating gonadal hormones between males and females. This can be accomplished by simply gonadectomizing (GDX) adult males and females. If the sex difference is no longer present after GDX, we know that the sex difference was caused by the differences in circulating hormones between males and females. Sex differences that disappear when circulating hormones are made equivalent between males and females are said to be due to “activational” effects of gonadal hormones.

If the observed sex difference persists even when males and females have the same circulating hormone exposure, the next logical step is to test whether the sex difference is influenced by developmental hormone exposure (i.e., “organizational” effects of hormones). Here, exposure to gonadal hormones during critical developmental periods causes permanent effects on the body, and these sex differences persist when adult hormones are made equivalent. The concept of the critical developmental window is actually quite tricky, as this window is not necessarily the same for every trait examined, and the window can extend from the prenatal into the postnatal period. Additionally, with respect to reproductive behavior, testosterone exposure during development performs both a masculinizing (organization of the neural control mechanisms for adult male sex behavior) and a defeminizing function (loss of ability to respond to the activational effects of ovarian hormones to induce female sex behavior); notably, testosterone may perform these organizational effects during different critical windows (see reviews [67,68]). Testing for organizational effects of hormones can be accomplished in a few different ways. One commonly used method is to treat females with a dose of testosterone similar to what males are normally exposed to during a critical developmental period; this critical period is typically thought to be right around the time of birth in rodents (but prenatally in some species; reviewed in [69]). If the females treated developmentally with testosterone are not significantly different from normal males, then the sex difference was due to organizational effects of hormones. Another method for testing for organizational effects of hormones is to remove the developmental testosterone exposure in males by GDX during the critical developmental period and determining whether these males are significantly different from normal females. However, this developmental GDX method is technically more challenging, as the procedure would have to take place prenatally, and it is difficult to know whether hormones were completely removed during the critical developmental window. There are several important questions to consider with studies aimed at manipulating hormone exposure during critical developmental windows: 1) Does incomplete masculinization or defeminization mean that the sex difference examined is not programmed by developmental hormone exposure or was the critical developmental window partially missed?, and 2) Is a single dose of testosterone enough for complete masculinization/defeminization or is prolonged exposure necessary? Notably, recent studies have identified puberty as an additional critical period for organizational effects of gonadal hormones (reviewed in [70]).

If the observed sex difference persists even after manipulating developmental hormone exposure, the next step is to test for potential effects of the sex chromosome complement. Genetic males have only one X chromosome and one Y chromosome, while genetic females have two X chromosomes. Thus, genes on the Y chromosome or gene dosage of the X chromosome could play a role in sexual dimorphism (reviewed in [71]). Even though researchers knew as early as the 1950s that the presence of the Y chromosome caused the undifferentiated gonads to develop into testes [72], work in the 1990s zeroed in on the SRY gene (*Sry* in mice) as being the testis-determining gene [73,74]. The testes in turn produce androgens to drive differentiation of the male internal and external genitalia. In the absence of the Y chromosome, and therefore lack of SRY/*Sry* gene product, the undifferentiated gonads develop into ovaries [72]. Since the testis-determining gene (*Sry*) is found on the Y chromosome, it is impossible to separate the potential role of sex chromosome complement from gonadal (and therefore, hormonal sex) in traditional wild-type mice, regardless of hormone manipulation. Thus, genetic manipulation has been used to engineer the four core genotypes (FCG) mice, in which *Sry* has been placed on an autosome after spontaneous deletion from the Y chromosome. Thus, genetic and gonadal sex are dissociated in the FCG mice, and the contribution of sex chromosome complement can be investigated independently (reviewed in [75]). Using the FCG mice, investigators can independently assess the contribution of sex chromosome complement, developmental hormone exposure, and adult circulating hormones to various observed sex differences. Importantly, to be able to probe for potential developmental hormone effects, all mice must be GDX several weeks prior to examination, such that any differences observed due to gonads are considered to be permanent changes due to hormone exposure during a critical period of development. A key strength of the FCG model is its ability to identify potential organizational hormone effects during the perinatal life and during puberty. Another mouse model that is useful for studying the contribution of sex chromosome complement to sex differences is the steroidogenic factor 1 (SF-1) KO mouse. SF-1 (encoded by the *Nr5a1* gene) is a transcription factor involved in the reproductive system and is normally expressed in the gonads, adrenal cortex, pituitary gland, and ventromedial nucleus of the hypothalamus [76,77]. SF-1 KO mice lack gonads and therefore also lack endogenous gonadal hormones during development and in adulthood; thus, SF-1 KO mice are useful to examine the effects of sex chromosome without the potential confounding effects of endogenous gonadal hormones [78-82].

Once sex chromosome complement has been identified as a contributing factor to the sex difference of interest, it is often important to determine whether the dosage of X chromosomes or the presence of the Y chromosome underlies the sex difference. To this end, researchers use the Y* mice, which have varying numbers of X and Y chromosomes, and Y chromosome consomic strains, in which the strains are genetically identical except for the Y chromosome (reviewed in [83]).

### Of mice and men: how do we investigate sex differences in mood disorders?

#### Humans

Our lab and others have reported numerous differences in the postmortem brains of MDD patients compared to healthy controls. The goal of these studies is to identify genes and proteins that are altered in the brains of MDD patients in order to identify factors that may cause MDD. Recent postmortem molecular studies [84-86] support the hypothesis of a deficit in inhibitory neurotransmission in MDD. Specifically, reduced expression of somatostatin (SST), a marker for inhibitory gamma-aminobutyric acid (GABA) neurons targeting pyramidal cell dendrites was observed in several brain regions in the corticolimbic network of mood regulation [sgACC [87], amygdala [86], and dorsolateral prefrontal cortex (DLPFC) [88]]. In concert, these findings suggest a GABA/SST-related cellular phenotype of reduced dendritic inhibition in depression. Using meta-analysis and meta-regression in eight human postmortem microarray studies in DLPFC, sgACC, and amygdala, we confirmed that SST is significantly decreased in subjects with MDD compared to matched controls and importantly, showed that the SST reduction in female MDD is significantly more robust than results in male MDD [89], together demonstrating a sexual dimorphism in reduced SST in MDD.

Another method that we have used in the human postmortem brain is gene co-expression analysis. Co-expression is defined as correlated gene expression across samples and has been shown to reflect shared gene function, including common regulation (e.g., hormones, transcription). The goal of these studies is to assess the broader biological context associated with our genes of interest. Using SST as our “seed” gene of interest, we identified GABA receptor signaling and mitochondrial dysfunction as the top canonical pathways represented by genes co-expressed with SST. Notably, this top 200 SST-co-regulated gene selection included GABA synthesizing enzymes glutamate decarboxylase 1 (GAD1; also known as GAD67) and GAD2 (also known as GAD65), hence confirming the functional relevance of an SST/GABA-related biological module [89]. Combined with our findings of a more robust reduction in SST in women with MDD, these gene co-expression studies suggest that more robust GABA-related deficits may characterize female MDD.

A major roadblock that we encounter when using human postmortem brains is that we often do not have blood samples from the same subjects, making circulating gonadal hormone analysis impossible. To partially circumvent this limitation, we have combined gene expression analysis and single nucleotide polymorphism (SNP) genotyping in the same subjects [i.e., expression quantitative trail loci (eQTL) study]. In these eQTL studies, we searched for SNPs (i.e., genetic polymorphisms) that are associated with either increased or decreased expression of our genes of interest. For instance, we performed a targeted eQTL study to test the hypothesis of X chromosome genetic contribution to SST, GAD1, and GAD2 gene expression. Even though the SST, GAD1, and GAD2 genes are not located on the X chromosome, we found several X chromosome SNPs associated with expression of these three genes; these results suggest the possibility of trans-regulation of SST, GAD1, and GAD2 by X chromosome-encoded factors [89]. Together, these correlative findings provide support for a contribution of genetic sex to sexual dimorphism in affect dysregulation in human subjects, potentially mediated by X chromosome trans-regulation of key GABA-related genes.

#### Mice

Although studies in the human postmortem brain have been highly informative in uncovering potential leads for the molecular mechanism underlying female vulnerability to MDD, complementary studies in mice are necessary to test for mechanisms underlying observed human sex differences. Importantly, we recently showed that the UCMS paradigm recapitulates the female vulnerability to MDD. Although both male and female UCMS-exposed mice developed elevated anxiety- and depressive-like behaviors compared to non-stressed controls, the chronically stressed females (which were freely cycling) exhibited a more robust elevation in behavioral emotionality compared to chronically stressed males [90], thus providing a needed assay to investigate the sexual dimorphic bases of human MDD. Here, “behavioral emotionality” or “emotionality” is defined as combined and measurable anxiety- and depressive-like behaviors in mice. Importantly, we did not find an interaction between UCMS exposure and sex, suggesting similar underlying mechanisms in males and females, but with additional factors at play in one or both sexes.

In a related experiment, we aimed to determine the potential contribution of developmental and adult hormone exposure to our observed sex difference in response to chronic stress exposure. To examine the potential developmental organizational role of hormones in establishing adult sex differences in emotionality, we tested the impact of neonatal testosterone exposure (a validated approach to developmentally “masculinize” the brain) [91-94] on adult emotionality in mice. We also examined potential activational hormone effects by comparing mice from each neonatal group that were GDX in adulthood and implanted with estradiol capsules or given sham surgery and blank implants. Results indicated that neonatal testosterone exposure partially masculinized UCMS-induced high emotionality of female mice; the females treated neonatally with testosterone displayed emotionality measures intermediate between normal males and females. Overall, we did not observe consistent activational effects of estradiol, although these studies were not designed to maximize these contrasts. Notably, other studies have reported effects of adult circulating hormones on emotionality (e.g., [95,96]). Indeed, Laplant and colleagues [97] reported that GDX of females prevents the pro-depressive-like effects of chronic stress. Our results in females treated neonatally with testosterone suggested that another factor, potentially sex chromosome complement, could influence the observed sex difference in emotionality. Although female mice in this study were treated neonatally with testosterone, mirroring the developmental testosterone exposure experienced by males, they were still genetically female for their entire lives; this suggests that genetic sex, regardless of developmental or adult hormone exposure, represents an additional factor contributing to adult emotionality. It is important to note that these females treated neonatally with testosterone did not have adult testosterone levels equivalent to a normal male. Thus, a “cleaner” model (e.g., FCG mice) is necessary to disentangle the potential effects of gonadal and genetic sex on emotionality.

Following up on the hint of a role for genetic sex, we used the FCG mice in a next set of experiments as a tool to separate the potential contributions of developmental hormone exposure, adult hormone exposure, and sex chromosome complement to adult emotionality. FCG mice were GDX as adults to remove endogenous gonadal hormones and implanted with testosterone-filled or blank capsules to also investigate the activational effects of testosterone. We then assessed anxiety-like behavior (using elevated plus maze and open field) under baseline (no stress) conditions and after exposure to UCMS. Under baseline conditions, the sex-related factor influencing emotionality was sex chromosome complement; however, the effect was in the opposite direction to what we had predicted based on the female vulnerability to mood disorders. Specifically, XY mice, regardless of gonadal sex or adult circulating testosterone treatment, exhibited increased anxiety-like behavior relative to XX mice. This sex chromosome effect was amplified after UCMS exposure. Additionally, we saw a potent effect of circulating testosterone to decrease anxiety-like behavior in UCMS-exposed mice, consistent with prior evidence in the literature [98]. Developmental hormone exposure had inconsistent effects on anxiety-like behaviors under both no stress and UCMS conditions. Although we reported a sex chromosome effect for the first time for anxiety-like behavior, a conventional interpretation in the sex difference field is that XY sex chromosome complement may exert a compensatory effect to reduce differences otherwise induced by circulating testosterone or vice versa [99]; indeed, FCG studies have reported similar opposing actions of XY and circulating testosterone [100,101]. Since “intact” male mice exhibit lower emotionality than females [90] and since we observed a more robust effect of circulating testosterone on lowering anxiety-like behaviors compared to the anxiogenic effect of XY genetic sex, circulating testosterone seems to “win out” in a normal male; the end result being lower anxiety-like behavior in males [89]. The robust behavioral findings and opposing effects of XY genetic sex and circulating testosterone demonstrate that both factors critically contribute to a dynamic equilibrium regulating adult anxiety-like behaviors.

To begin to search for the molecular underpinnings of the opposing effects of male sex chromosome complement and circulating testosterone on anxiety-like behavior, we examined expression of several mood-related genes in the frontal cortex of FCG mice. Specifically, we began by examining expression of several genes related to GABA, serotonin, and dopamine signaling, as candidate systems implicated in mood disorders. Several studies suggest impaired excitation/inhibition balance in mood disorders, potentially mediated by decreased GABA inhibition [84,86-88,102-106]. Additionally, results also suggest problems with slow-acting serotonin and dopamine neuromodulatory systems in mood disorders [107-115]. Interestingly, results showed that the sex-related factor that had the strongest effect on expression of these mood-related genes was sex chromosome complement. Overall, mice with XY sex chromosome complement tended to have lower expression of these GABA-, serotonin-, and dopamine-related genes compared to XX mice. These gene expression findings correlated nicely with our finding that XY mice also had elevated anxiety-like behavior. Developmental hormone exposure resulted in varied effects: mice with male hormone exposure during development had higher expression of GABA-related genes but lower expression of serotonin- and dopamine-related genes. Adult testosterone exposure exhibited inconsistent effects [116]. Together, these studies provided some molecular support to the behavioral studies investigating the contribution of XY sex chromosome complement to adult behavioral emotionality.

## Conclusions

**Figure 1:**
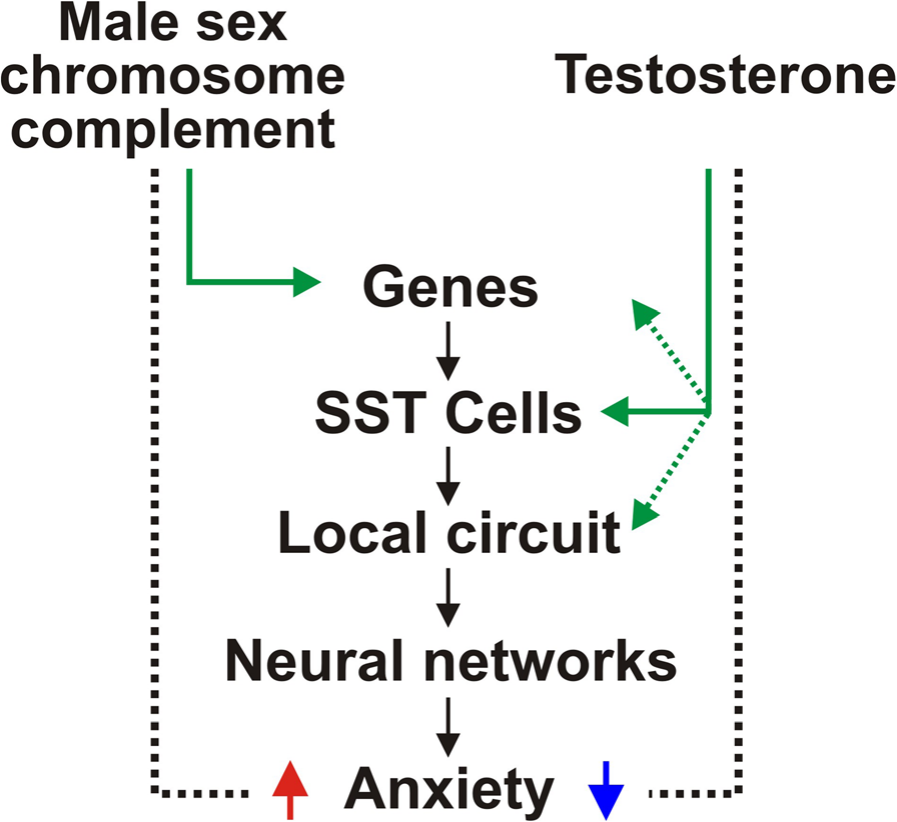
**A novel model depicting a dynamic balance between sex chromosome complement and circulating testosterone on anxiety (black dotted line).** A conventional interpretation in the sex difference field is that XY sex chromosome complement may exert a compensatory effect to reduce differences otherwise induced by circulating testosterone or vice versa. Data outlined in this review suggests that this is also the case for the regulation of mood and anxiety-like behaviors. Specifically, while male sex chromosome complement causes an increase in anxiety-like behavior, this effect is opposed by testosterone’s antianxiety effect. While sex chromosome complement appears to be acting via control of gene expression, we hypothesize that testosterone affects the activity of certain inhibitory GABA cell types (e.g., SST cells).

There is clear evidence that women are more vulnerable to develop mood disorders compared to men. This sex difference seems to have a biological basis, as we have found sex differences in expression of mood-related genes in the brains of depressed subjects. Our work thus far suggests that a dynamic equilibrium exists between the effects of male sex chromosome complement to increase anxiety, which is opposed by the antianxiety effects of male circulating testosterone exposure. Figure 1 provides a schematic summarizing our interpretation of the findings described in the previous sections. Specifically, our work in humans and in mice shows that sex chromosome complement influences expression of SST and other GABA-related genes [89,116]. Our mouse studies also show that while testosterone has a potent effect of decreasing anxiety-like behavior, it does not seem to be doing so via effects on GABA-, serotonin-, or dopamine-related gene expression [89,116]. We hypothesize that testosterone acts to oppose the pro-anxiety effects of male sex chromosome complement by affecting the function of SST cells and/or the function of the local cortical microcircuitry (Figure 1). Finally, we believe that preliminary studies using appropriate mouse models, with consideration of trait and state, as well as the multiple dimensions of mood-related behaviors, can provide a framework to systematically dissect the biological underpinnings of sex differences in mood in humans.

## Abbreviations

DLPFC: dorsolateral prefrontal cortex
eQTL: expression quantitative trait loci
FCG: four core genotypes
GABA: gamma-aminobutyric acid
GAD1: glutamate decarboxylase 1
GAD2: glutamate decarboxylase 2
GAD65: glutamate decarboxylase 65
GAD67: glutamate decarboxylase 67
GDX: gonadectomize
HPA: hypothalamic pituitary adrenal
MDD: major depressive disorder
SF-1: steroidogenic factor 1
sgACC: subgenual anterior cingulate cortex
SNP: single nucleotide polymorphism
SSRI: selective serotonin reuptake inhibitor
SST: somatostatin
UCMS: unpredictable chronic mild stress
